# Parents’ Intentions and Associated Factors to Vaccinating Their Children Aged 12–17 Years with COVID-19 Vaccines: A Cross Sectional Study

**DOI:** 10.3390/vaccines10060912

**Published:** 2022-06-08

**Authors:** Osama Al-Wutayd, Manal Al-Batanony, Nehad Badr, Sally Abdelwanees

**Affiliations:** 1 Department of Family and Community Medicine, Unaizah College of Medicine and Medical Sciences, Qassim University, Unaizah 56219, Saudi Arabia; ma.albatanony@qu.edu.sa; 2 Department of Public Health and Community Medicine, Menoufia Faculty of Medicine, Menoufia University, Shebin Al-Kom 6131567, Egypt; nihad.ali@med.menofia.edu.eg (N.B.); sally.hasan.12@med.menofia.edu.eg (S.A.)

**Keywords:** parents, COVID-19 vaccination intention, children aged 12–17 years

## Abstract

No available vaccine against COVID-19 had yet been proven for 12–17-year-olds in Egypt during the study period. This is the first study to assess Egyptian parents’ intentions and associated factors in relation to vaccinating their children with COVID-19 vaccines. A cross-sectional study using a questionnaire was conducted between 17 October and 17 November 2021, via social media platforms. The target group was parents with children aged 12–17 years. Parents’ intention to vaccinate their children and factors associated with vaccinating their children, reasons for not intending to vaccinate their children, and circumstances whereby the parents would change their mind were recorded. Among the 1458 parents recruited, 65.6% were planning to vaccinate their children. The main concerns were fear of the vaccine’s side-effects (68.3%) and conspiracy theories (18%). The factors associated with parents’ intention to vaccinate their children were mother’s older age (40–49 years: aOR = 1.45, 95% CI = 1.05–1.99; ≥50 years: aOR = 2.09, 95% CI = 1.16–3.75), high family income (aOR = 1.99, 95% CI = 1.41–2.79), and children with a history of chronic conditions (aOR = 2.02, 95% CI = 1.25–3.25), while higher mother’s education level was negatively associated (aOR = 0.41, 95% CI = 0.27, 0.64). A comprehensive health education program regarding vaccinating children with COVID-19 vaccines is highly recommended for parents, particularly for young and highly educated mothers, to enhance children vaccination rate when the vaccine becomes available.

## 1. Introduction

The safety and effectiveness of vaccines is, undoubtedly, a sensitive topic of great public concern, and it has been well established and proven that immunizations is among the most effective community-applied measures for combating the spread of many viral illnesses, decreasing disability, protecting vulnerable groups from severe illnesses, and saving lives [1]. Severe acute respiratory syndrome coronavirus 2 (SARS-CoV-2), or coronavirus, was identified in January 2020 before a pandemic spread to over 170 countries [2]. The American Academy of Pediatrics reported that, since the beginning of the pandemic, approximately 12.3 million children have tested positive for COVID-19, and more than 2.9 million cases were added in the last four weeks [3]. The Egyptian confirmed cases of COVID-19 reported to the WHO were estimated to be 513,944, and deaths were 24,718 in the period between 3 January 2022 and 31 May 2022 [4]. However, there are no available data about children affected by COVID-19. It seems that the majority of children are not affected, also taking in consideration many other factors, such as subpopulations of children at greater risk, the actual attributable risk for severe illness from COVID-19 (e.g., coexisting infections), and the role of children in transmitting infection to their relatives [5]. A multicenter study in Egypt reported that 40 cases of COVID-19 with mean age of 9.4 years were admitted in hospitals with no significant morbidities or mortality [6]. Vaccine hesitancy (VH) is defined as “a behavior influenced by a number of factors including issues of confidence (do not trust a vaccine or a provider), complacency (do not perceive a need for a vaccine or do not value the vaccine), and convenience (access) [7]. However, it cannot be denied that different degrees of concern exist among different populations towards children being vaccinated with COVID-19 immunization shots for a number of reasons [8]. Previous studies concluded that parents’ strongest reasons for refusing to vaccinate their offspring were centered on the vaccine’s side-effects [9,10], safety [11,12], and necessity [10]. A recently published study in the U.S.A. reported that one in five parents were hesitant towards the COVID-19 vaccine [12]. VH is unequally distributed among people. In many situations, parents and caregivers are well educated and informed about the importance of vaccines, while others have received little communication and solid scientific information, which causes a barrier to improving vaccination coverage [9]. Because parents make decisions about their children’s lives, healthcare, personal beliefs, and lifestyle, the trustworthiness and accuracy of the information they receive affect their ability to make decisions about their child’s vaccines [12]. Vaccines against COVID-19 for children are now a reality, and there have been calls for an expansion of vaccine trials to cover children [13]. In May 2021, the U.S. Food and Drug Administration and the European Union granted emergency approval for a COVID-19 vaccine for use in children aged 12 to 15 [14]. In December 2020, the Pfizer-BioNTech and Moderna mRNA vaccines against COVID-19 were approved and recommended by Health Canada for use in the population aged 16–18 years. After clinical trials, Health Canada extended the approval for the provisional appointment of the Pfizer-BioNTech vaccine to cover children aged 12–15 years (on 5 May 2021) and the Moderna COVID-19 vaccine to children aged 12–17 years (on 27 August 2021). Both vaccines are administered in two intramuscular doses three and four weeks apart, respectively [15]. The five available vaccines approved for use in Egypt are Sputnik V, Sinopharm, AstraZeneca, Johnson& Johnson, and Sinovac [16]. Egypt had fully vaccinated 34.5 million people as of May 2022, which is a significant figure, but more needs to be done. Vaccination was not available in Egypt until recently for children under the age of 18 years. Many families still expressed vaccine hesitation for themselves and their children after the Pfizer immunization was approved for children over the age of 12 with parental consent. Vaccination rates for youngsters were projected to be quite low in remote Egyptian areas [17]. In Egypt, during the study period, no available vaccine against COVID-19 had been approved for the 12–17-year age group. As possible fears over and rejections of COVID-19 vaccines need to be investigated, to the best of our knowledge, parents’ acceptance of their children’s vaccination against COVID-19 in Egypt has not been reported. The aim of this study was to highlight parents’ intentions and associated factors in relation to vaccinating their children with COVID-19 vaccines.

## 2. Methods

### 2.1. Study Design and Sampling

A cross-sectional study was conducted among Egyptian parents of children aged 12–17 years from 17 October to 17 November 2021. Parents who met the inclusion criteria and represented a convenient non-probability sample were our target group. Inclusion criteria were set for parents of one child or more, aged from 12 to 17 years, who lived in Egypt, could read and write, and had a social media account.

### 2.2. Questionnaire and Data Collection

Via a Google Form, a self-administered web-based questionnaire written in Arabic was used. The investigators created this questionnaire based on recent literature [10,11,18,19]. A questionnaire link was generated and distributed on different social media platforms (Facebook, WhatsApp). The questionnaire consisted of an interface (containing the title, study aim, voluntary responses, confidentiality statement, the time it would take to complete the questionnaire, and whether or not he/she would consent to participate). The first part consisted of sociodemographic data, including age, sex, residence location, education level, and occupation of the mother and father, monthly family income and presence of any chronic comorbidities (lung diseases, cancer, blood disorders, diabetes mellitus, and obesity) in their children. The second part consisted of the reasons for the parents who did not intend to vaccinate their children; the circumstances that would enable them to allow the children to have a vaccine were also asked. English was initially used to formulate the questionnaire, which was carefully revised by experts (3 epidemiologists) for content validity. To test the language capability of the questionnaire, it was translated into Arabic and then back to English. The Arabic translation was further reviewed before questionnaire distribution. A pilot study of 30 parents was performed for face validity, and its results were excluded from the study. The Cronbach’s Alpha was 0.72, indicating acceptable internal consistency. The sample size was calculated using the EPI 7™ software program. Assuming adequate power (80%), the margin of error (5%), and considering the prevalence of parents’ intention to have their children vaccinated was 64% [17]. The minimum sample size was 460 after assuming a non-response rate and/or incomplete responses of 30%. Approval to perform this study was granted by Menoufia Faculty of Medicine Research Ethics Committee on 12 January 2021 (Reference No.2/2022COM), and online Informed consent was obtained from each participant prior to the study commencement. We approached 2791 subjects, and 1458 returned the questionnaire with complete responses, while the rest did not agree to participate or submitted incomplete responses; thus, the response rate was 52%.

### 2.3. Data Management and Analysis Plan

Stata version 17 was used for data analysis. The data are presented as frequency and percentage for categorical variables. To assess the risk association of independent factors with the dependent one (parents’ intention to vaccinate their children), a multiple logistic regression was performed, and only variables with *p* value < 0.25 in simple logistic regression were selected in the multivariable analysis. Crude and adjusted odds ratios in simple and multiple logistic regression analysis models are reported, respectively. A two-sided *p*-value ≤ 0.05 was considered strong evidence against the null hypothesis.

## 3. Results

Of the 1458 questionnaires completed in this study, 66.5% were by mothers. The age of mothers and fathers was predominantly 40–49 years (48.2% and 44.3%, respectively). The majority of parents had a bachelor’s degree or higher (89% and 92.1% for fathers and mothers, respectively). Most of the parents were employed (81.6% and 92.1% for mothers and fathers, respectively), urban residents (87.4%), and had high family income (84.2%), and most of the children had no chronic comorbidities (93.1%). Of the parents, 65.6% fully intended to allow their children to receive the vaccine when it would become available, while 34.4% were hesitant (Table 1). Among those who did not intend to vaccinate their children, the main concern was fear of side effects (68.3%) and conspiracy theories (18%). Moreover, on asking the same group about the circumstances in which they might allow their children to be vaccinated, the need for school attendance (28.3%), advice from a trusted physician (19.8%), sufficient studies approving safety and effectiveness (19.8%), and vaccination becoming a governmental commitment (11%) were highlighted (Table 2). In simple logistic regression, older mother’s age (40–49 years: cOR = 1.54, 95% CI = 1.22–1.94; ≥50 years: cOR = 2.46, 95% CI = 1.56–3.86), older father’s age (≥50 years: cOR = 1.75, 95% CI = 1.29–2.37), and families with high income (cOR = 1.66, CI 95% = 1.24–2.20) were significantly associated with a higher intention of the parents to vaccinate their children. In contrast, a higher level of mother’s education (bachelor’s degree or higher, cOR = 0.64, 95% CI = 0.45–0.93) was negatively associated with parents’ intention to vaccinate their children. Multivariable analysis revealed that older mother’s age (40–49 years: aOR = 1.45, 95% CI = 1.05–1.99; ≥50 years: aOR = 2.09, 95% CI = 1.16–3.75), high family income (aOR = 1.99, CI 95% = 1.41–2.79) and children with history of chronic conditions (aOR = 2.02, 95% CI = 1.25–3.25) were factors associated with parents’ intention to vaccinate their children. On the other hand, mother’s bachelor’s degree or higher (aOR = 0.41, 95% CI = 0.27–0.64) was negatively associated with parents’ intention to vaccinate their children (Table 3).

## 4. Discussion

The current study revealed that around two-thirds of the participating parents were willing to allow their children to be vaccinated against COVID-19, whereas around one-third were hesitant. This may be due to the increase in the number of cases and deaths during the study period. This finding is consistent with several studies [20]. In New York City, 61.9% of parents were planning to vaccinate their children, whereas 14.8% completely refused, and 23.3% were uncertain of their opinion towards the vaccine [21]. In Korea, 64.2% of parents intended to have their children vaccinated [22]. Moreover, Chinese parents’ acceptance of vaccinating their children under 18 years was 72.6% [23]. The prevalence of parents’ reluctance to vaccinate their children in Turkey was 66.1% [24]. In Arab countries, parents’ intention ranged from 30% in Jordan [25] and 38% in Iraq [26] to 75% in Saudi Arabia [27]. In the current study, the main concerns of the parents to vaccinate their children were concerns over the vaccine’s side effects, worries about conspiracy theories regarding the vaccine, and beliefs that the vaccine had no protective value. Adverse side effects following vaccination were reported as the main reason for parents’ vaccine hesitancy in several previous studies: 84.1% in Korea [22], 61.5% in Boston [28], and 46.8% in Romania [10]. A report by Imperial College London on global attitudes toward COVID-19 vaccination demonstrated that both side effect concerns and insufficient testing data during the vaccine approval process were the cornerstones against vaccine uptake [29]. Additionally, the lack of need for children to be vaccinated (49.5%) was reported as an important reason for vaccine unacceptability among Romanian and American parents [10,21]. Unlike infant vaccines that are created over time and acquire parents’ trust over years of usage, the COVID-19 vaccines were produced in a very limited time; as a result, people may be skeptical of their usefulness in preventing COVID-19-related harm, particularly in youngsters. The novelty of COVID-19 vaccines is among the most common reasons for caregivers to refuse vaccination for their children [30,31]. In the present study, using multivariable analysis, older mother’s age, high family income, and children with chronic conditions appeared to be the main predictors for parents’ intention to vaccinate their children. However, a higher level of mother’s education (bachelor’s degree or higher) was negatively associated with parents’ intention to vaccinate their children. In accordance, recent research in France among working-age individuals found that a younger mother’s age was a significant predictor for vaccine hesitancy and refusal [32]. Another recent study in Romania found a significant positive correlation between older-aged parents and planning for vaccinating their teenage children [9]. Other published studies found that low socioeconomic status was a predictor for vaccine hesitancy among parents [22,33]. In contrast with our result, the likelihood of children’s vaccination was found to be greater among parents with a bachelor’s degree or higher in Romania, Germany, and Italy [10,34,35]. This contradiction could refer to Western populations generally having a higher education level than our study group in Egypt. Moreover, the highly educated parents in the current study had a lower confidence level in vaccine safety as well as a higher belief in exaggerated policy measures beside conspiracy theories. Commitment to school entrance, advice from a trusted physician, and approval of the vaccine’s safety and effectiveness by enough studies were the main circumstances that could lead the hesitant parents in this study to change their mind toward vaccinating their children. In line with this finding, Tsai et al. [11] concluded that recommendation from a trustworthy physician plays a key role in convincing caregivers of the importance of vaccinating their children against COVID-19. Some notable limitations are present. Selection and report bias cannot be ruled out, as the study group was enrolled through online request, which was the approved method for recruiting participants during the COVID-19 pandemic. The study may have been subject to social desirability and recall bias because the data were self-reported by the parents. In addition, authenticity of the responses may not be certain, since the survey was administered online and was web-based. In addition, the concordance between the parents’ vaccination status and their intention to vaccinate their children was not asked, although until now, the utilization of vaccination has not been a social norm in Egypt. Finally, the study design was cross-sectional, which limits the inference on causality of the associated factors.

## 5. Conclusions

A comprehensive and tailored community awareness children vaccination program about COVID-19 is warranted. Convincing parents, particularly, young and highly educated mothers, to vaccinate their children against COVID-19, once the vaccine becomes available in Egypt, requires particular attention, and efforts should begin now. Such awareness can be obtained through home visits as part of a “door-knocking” campaign to talk about COVID-19 vaccines.

## Figures and Tables

**Table 1:** Sociodemographic data of the study participants according to their intention to vaccinate their children.

Sociodemographic Data	Total	Intent to Vaccinate n (%)	Hesitant to Vaccinate n (%)
Total	1458	957 (65.6)	501 (34.4)
Respondent parent			
Mother	969 (66.5)	625 (64.5)	344 (35.5)
Father	489 (33.5)	332 (67.9)	157 (32.1)
Mother’s age (years)			
<30	71 (4.9)	42 (59.1)	29 (40.9)
30–39	557 (38.2)	330 (59.2)	227 (40.8)
40–49	702 (48.2)	485 (69.1)	217 (30.9)
≥50	128 (8.8)	100 (78.1)	28 (21.9)
Father’s age (years)			
30–39	327 (22.4)	201 (61.5)	126 (38.5)
40–49	645 (44.3)	398 (61.7)	247 (38.3)
≥50	486 (33.3)	358 (73.7)	128 (26.3)
Mother’s education			
Secondary school or below	161 (11)	119 (73.9)	42 (26.1)
Bachelor’s degree or higher	1297 (89)	838 (64.6)	459 (35.4)
Father’s education			
Secondary school or below	115 (7.9)	71 (61.7)	44 (38.3)
Bachelor’s degree or higher	1343 (92.1)	886 (66)	457 (34)
Mother’s occupation			
Unemployed	268 (18.4)	169 (63.1)	99 (36.9)
Employed	1190 (81.6)	788 (66.2)	402 (33.8)
Father’s occupation			
Unemployed	113 (7.8)	71 (62.8)	42 (37.2)
Employed	1345 (92.2)	886 (65.9)	459 (34.1)
Residence			
Urban	1274 (87.4)	844 (66.3)	430 (33.7)
Rural	184 (12.6)	113 (61.4)	71 (38.6)
Family income (EP/month)			
<5000	230 (15.8)	128 (55.7)	102 (44.3)
≥5000	1228 (84.2)	829 (67.5)	399 (32.5)
Children with history of chronic co-morbidities			
No	1357 (93.1)	884 (65.1)	473 (34.9)
Yes	101 (6.9)	73 (72.3)	28 (27.7)

**Note:** EP = Egyptian pounds.

**Table 2:** Reasons for parents who did not intend to vaccinate their children and circumstances whereby they might allow them to receive a vaccine.

	Total (n = 501)
Reasons for Non-Intentional Attitude	n (%)
Fear of the vaccine’s side effects.	342 (68.3)
The vaccine has no protective value against the disease.	42 (8.4)
My son/daughter follows the protective precautions (hand washing, masks, and gloves), so there is no need for vaccination.	9 (1.8)
My son/daughter is afraid of or dislikes injections.	10 (2.0)
I do not rule out a conspiracy theory regarding COVID-19 vaccination.	90 (18.0)
My son/daughter is young and healthy, so there is no need for vaccination.	8 (1.6)
Additional reasons (no = 56):	
- I cannot afford it.	14 (25%)
- I worry about mortality/morbidity cases after vaccination.	14 (25%)
- There have not been enough studies about the vaccines.	16 (28.6)
- The vaccine is imported and not trusted.	12 (21.4)
The circumstances whereby parents may allow their children to receive the vaccine	
	**(n = 501)**
Response	**n (%)**
I will not allow my son/daughter to receive the vaccine under any circumstance.	100 (20.0)
If a trusted physician advises me to do so.	99 (19.8)
If vaccination becomes necessary for school attendance.	142 (28.3)
If vaccination becomes a governmental commitment.	55 (11.0)
If the vaccines are approved by enough studies for safety and efficacy.	99 (19.8)
If another route of administration other than injection is proven.	6 (1.2)

**Table 3:** Crude and adjusted odds ratio of factors associated with the intention of parents to vaccinate their children.

Characteristics	Crude Odds Ratio		Adjusted Odds Ratio	
	cOR (95% CI)	*p*-Value	aOR (95% CI)	*p*-Value
Respondent parent				
Mother	Reference		Reference	
Father	1.16 (0.92, 1.47)	0.198	1.10 (0.87, 1.40)	0.424
Residence				
Urban	Reference			
Rural	0.81 (0.59, 1.12)	0.197	0.94 (0.66, 1.33)	0.716
Mother’s age (years)				
<30	0.99 (0.60, 1.65)	0.988	1.07 (0.62, 1.84)	0.812
30–39	Reference		Reference	
40–49	1.54 (1.22, 1.94)	<0.001 *	1.45 (1.05, 1.99)	0.024 *
≥50	2.46 (1.56, 3.86)	<0.001 *	2.09 (1.16, 3.75)	0.014 *
Father’s age (years)				
30–39	Reference		Reference	
40–49	1.01 (0.77, 1.33)	0.943	0.91 (0.66, 1.26)	0.569
≥50	1.75 (1.29, 2.37)	<0.001 *	1.15 (0.73, 1.80)	0.551
Mother’s education				
Secondary school or below	Reference		Reference	
Bachelor’s degree or higher	0.64 (0.45, 0.93)	0.020 *	0.41 (0.27, 0.64)	<0.001 *
Father’s education				
Secondary school or below	Reference			
Bachelor’s degree or higher	1.20 (0.81, 1.78)	0.360		
Mother’s occupation				
Employed	Reference			
Unemployed	0.87 (0.66, 1.15)	0.325		
Father’s occupation				
Employed	Reference			
Unemployed	0.88 (0.59, 1.30)	0.513		
Family income (EP/month)				
<5000	Reference		Reference	
≥5000	1.66 (1.24, 2.20)	0.001 *	1.99 (1.41, 2.79)	<0.001 *
Children with history of chronic co-morbidities				
No	Reference		Reference	
Yes	1.39 (0.89, 2.19)	0.147	2.02 (1.25, 3.25)	0.004 *

**Note:** EP = Egyptian pound, * *p* value < 0.05

## Data Availability

The data presented in this study are available on request from the corresponding author.
